# Analysis of pressure-bearing performance and optimization of structural parameters of the slip in a compression packer

**DOI:** 10.1177/0036850419881106

**Published:** 2019-10-21

**Authors:** Yang Tang, Peng Sun, Guorong Wang, Wang Li

**Affiliations:** 1 School of Mechatronic Engineering, Southwest Petroleum University, Chengdu, China; 2 State Key Laboratory of Oil and Gas Reservoir Geology and Exploitation, Southwest Petroleum University, Chengdu, China; 3 Key Laboratory of Oil and Gas Equipment, Ministry of Education, Southwest Petroleum University, Chengdu, China; 4 Southern Marine Science and Engineering Guangdong Laboratory (Zhanjiang), Zhanjiang, China

**Keywords:** Slip, packer, pressure-bearing performance, finite element analysis, structural parameter optimization

## Abstract

In drilling and completion operations, a packer is a downhole tool to seal the annular space between various sizes of string and borehole. As a key component of packer, slip plays a role of fixed support in the process of setting, thus ensuring a long-term stable sealing of the packer. Aiming at the problem of damage caused by casing pressure on the slip after setting, based on the stress analysis of slip during setting, the slip material with hardness lower than casing was selected, and its finite element analysis model was established. Then, the pressure-bearing law of the slip teeth under three tooth parameters was analyzed. The results of analysis were as follows. With the increase of the number of slips or the distance between teeth or tooth top angle, the strain of slips decreased, but the bending moment of slips increased. At the same time, orthogonal test method was used to optimize the parameters of the slips. The conclusion is that the tooth top angle is 90 degrees, the distance between teeth is 6 mm and the number of teeth is 16, which is the best combination of compressive effect. It will provide a reference for improving the long-term effectiveness of the packer seal in drilling and completion engineering.

## Introduction

In the process of oil and gas mining, the requirements for underground tools are becoming higher because of the increase in mining difficulty and the improvement in technology.^1,2^ In the anchoring mechanism of packer, slip has the function of supporting the packer and fixing the rubber cylinder. Its performance directly affects the working life of the packer.^3^ In practical engineering applications, serious damage of slips will lead to loss of sealing, slippage, and leakage. Thus, the cost of mining will be increased and the mining efficiency will be affected.^4,5^ Therefore, it is necessary to further study the pressure-bearing performance of slip.

There are few studies on the pressure-bearing performance of slips at home and abroad. Tong et al.^6^ analyzed stress distribution and strength of RTTS packer slips by means of the stress analysis method of elastic mechanic wedge. Tang et al.^7^ adopted the finite element analysis (FEA) method to optimize slip structure on the premise of ensuring sufficient anchorage force after the slip was seated. Chao et al.^8^ applied FEA software to simulate stress of clamping and rotating casing string with different types of teeth, through the modeling of dynamic slip and the FEA. Gao et al.^9^ discussed the relationship between several important geometric parameters of slip tooth and tightening effect. Sun et al.^10^ have studied the interaction between slip and bushing by theory and FEA method. Wang et al.^11^ adopted the FEA method and three-dimensional photo elastic technology to analyze the contact stress of packer slips.

However, because the mechanical parameters of slips are larger than that of casing, the existing research mainly focuses on the damage effect of slips on casing. Few studies have been focused on the pressure-bearing performance of slips. None of these studies have considered the factors affecting the pressure-bearing performance of slips comprehensively. In this article, we will take a compression packer slip as the object of analysis to reduce mechanical properties of its material as the premise. Then, we will use the FEA method to analyze slip pressure-bearing performance change rule by considering tooth number of slip, tooth space, tooth top angle, and other factors. Orthogonal experimental method is used to optimize design parameters of the slip, to reduce its damage. This study will provide a theoretical basis for further analysis of pressure-bearing performance of the slip and design of new slips.

## Force analysis of the packer slip in the sealing process

### Sitting seal principle of the compression packer

**Figure 1.**
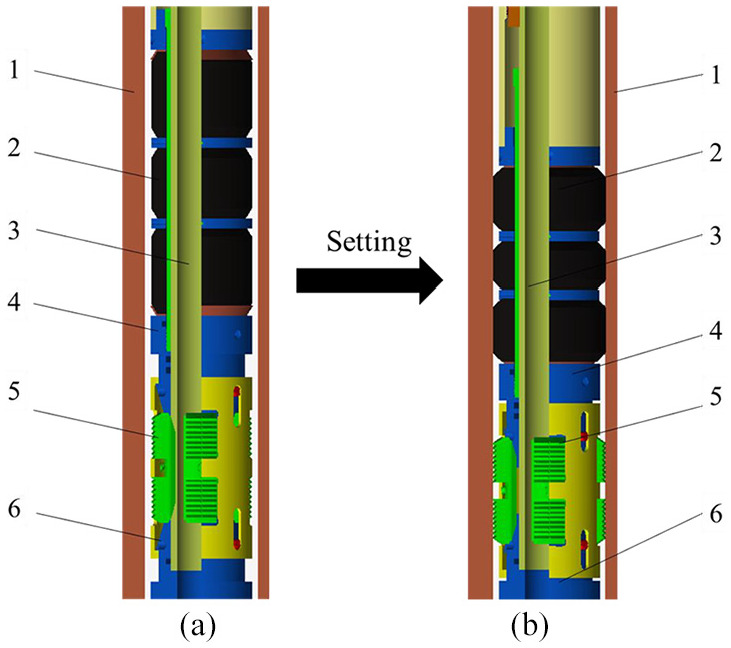
A schematic diagram of seal process of a compression packer: (a) the packer state before sealing and (b) the packer after sealing. 1: casing; 2: rubber cylinder; 3: central tube; 4: upper cone; 5: slip; 6: lower cone.

The sealing force of the compression packer is hydraulic pressure. It is produced by its hydraulic sealing tool. First, the position of the packer is estimated according to the number of downloaded columns. Then, the sealing mechanism is connected and lowered to the target section (Figure 1(a)).^12^ The inner flow of the packer is blocked by a steel ball, because density of the ball is larger than density of the downhole mixture. The ball can reach the inner circular cone of the short section of the packer by gravity settlement. Then, the fluid pressure is injected into tubing. When pressure being calibrated, the pressure is stabilized for a period of time. And the hydraulic pressure pushes piston, slide sleeve, lock ring, seal, slip, and cone to be moved down, so that the seat clipper is cut off. The connection between locking housing and releasing hand claw is disconnected. The shell joint of the hydraulic chamber drives outer casing to move downward to push the upper cone, so that the slips are pushed out. The slips are gradually opened and snare on the inner wall of casing. The locking ring moves downward along the locking device, and the slips are fixed on sleeve to achieve the anchoring effect. Other sealing tools have not yet reached the limit stroke, and still need to continue to move. Then, they are extruded and expanded to seal them (Figure 1(b)).^13^

### Force analysis of compression packer slip

According to the anchoring principle of compression packer slip, considering the nonlinearity in its anchoring process, the following simplifications were made.^14^

1. As slip was the same as the casing anchoring principle around the center tube, only a single slip was analyzed. The model had four parts: slip, cone, casing, and central tube. The inner surface of the slip was in contact with the cone, and the outer surface was in contact with the casing.^15^
2. As the slip was distributed around the central tube, it was not axisymmetric. But the center tube, the casing, and the cone were axisymmetric figures.^16^ In order to improve the calculation speed, it was set to two-dimensional model analysis and neglected fine features on slip and cone. The casing size was determined by American Petroleum Institute standard, which was 139.7 mm, inner diameter 121.4 mm, and 9.17 mm wall thickness.

**Figure 2.**
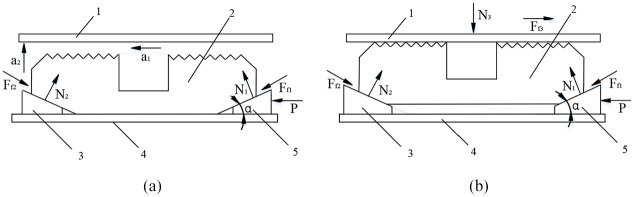
Force condition of slip before and after occlusion with casing during packer setting: (a) the state before occlusion and (b) the state after occlusion. 1: casing; 2: slip; 3: lower cone; 4: central tube; 5: upper cone.

The stress analysis of slip before and after snapping sleeve was carried out during the sealing process, and the stress state of slip is shown in Figure 2.

In Figure 2(a), the slip was accelerated to left by the force of upper cone at the beginning of seating, and then accelerated upward with lower cone, so the slip was subjected to horizontal force

(1) $$ma_{1}=\left(F_{f1}-F_{f2}\right)\cos\alpha +\left(N_{1}-N_{2}\right)\sin\alpha$$

In equation (1), *m* is the slip quality; $a_{1}$ is the acceleration to the left of slip; $N_{1}$ is the pressing force of upper cone acting on the cone of slip; $N_{2}$ is the compaction force of lower cone acting on the cone of slip; $F_{f1}$ is the friction between cone and upper cone of slip; $F_{f2}$ is the frictional force between cone and lower cone of slip; and α is the slip wedge angle.

Stress on the slips in the vertical direction

(2) $$ma_{2}=\left(N_{1}+N_{2}\right)\cos\alpha -\left(F_{f1}+F_{f2}\right)\sin\alpha$$

where $a_{2}$ is the acceleration to upward of slip.

From the law of friction

(3) $$F_{f1}=fN_{1}$$

(4) $$F_{f2}=fN_{2}$$

where *f* is the friction factor between cone and cone of slip.

In Figure 2(b), the slip was occluded on sleeve after the seating completed, and then the slip was subjected to horizontal force

(5) $$F_{f3}=\left(F_{f1}-F_{f2}\right)\cos\alpha +\left(N_{1}-N_{2}\right)\sin\alpha$$

(6) $$F_{f3}=\frac{Q_{z}}{n}$$

where $F_{f3}$ is the friction between slip and casing; $Q_{z}$ is the axial loading of packer; and *n* is the number of slips.

Stress on the slips in the vertical direction, equation (7), can be obtained by

(7) $$N_{3}=\left(N_{1}+N_{2}\right)\cos\alpha -\left(F_{f1}+F_{f2}\right)\sin\alpha$$

From the law of friction

(8) $$F_{f3}=f*N_{3}$$

where $N_{3}$ is the pressure of casing acting on slip; and f* is the friction factor between slip and casing.

## Establishment of the FEA model of the slip

### Acquisition of material parameters of slip

**Figure 3.**
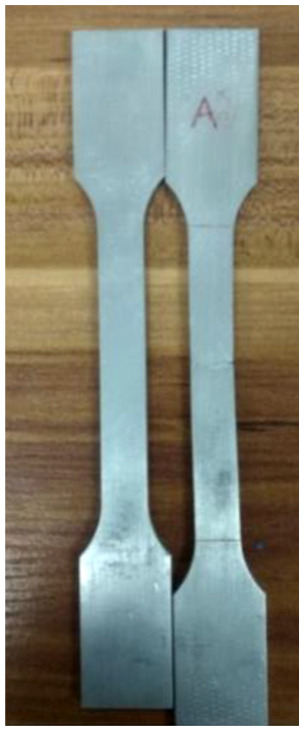
Material sample of slip.

In order to choose the material which was more suitable for pressure-bearing performance of slip, mechanical properties of the slip had been reduced to meet damage condition of casing. Soluble material was selected for the tensile test.^17^ It was mainly for determining elastic modulus, Poisson’s ratio, yield strength, and tensile strength of the material.^18^ As shown in Figure 3, specimen’s head shape and size should be adapted to clamping of the test machine. The specimen was in the shape of a plate, and the size was 12.51 mm × 3.4 mm.

**Figure 4.**
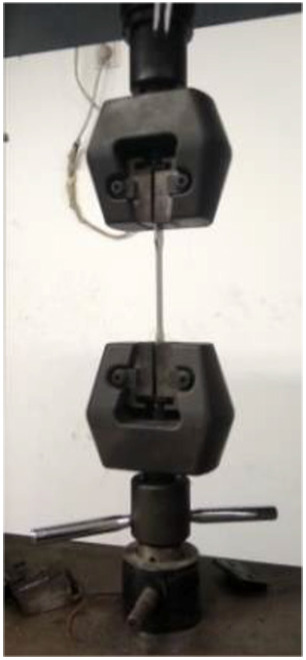
Tensile test machine.

**Figure 5.**
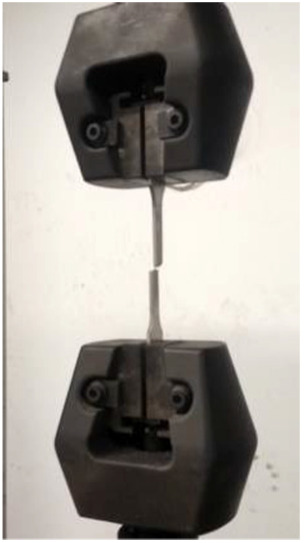
Fracture of plate after test.

From Figures 4 and 5, the plates were sandwiched between the top and bottom two fixtures, respectively, in the test. Then, the test machine had been restarted for continuous pressure until the specimen was broken, and the lower end of the force sensor was connected with upper fixture.^19^ During the test, the force of the sample was changed into an electrical signal through the force sensor, and input to the acquisition control system. As the sample was subjected to pressure, the distance between two jigs increased correspondingly. The electrical signal collected by the encoder was input to the acquisition control system by tracking the encoder. Data were saved by measurement and control software.^20^

**Figure 6.**
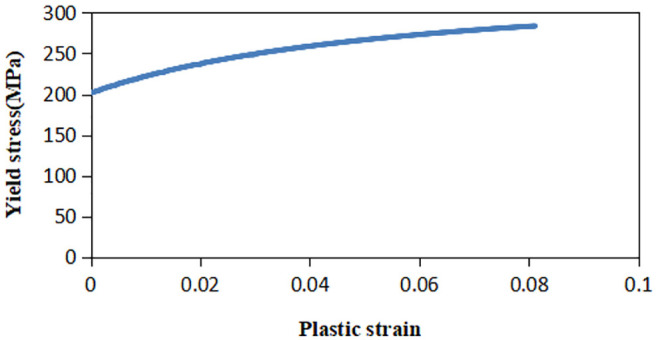
Relationship between yield stress and plastic strain of slip experimental material.

The relationship between yield stress and plastic strain of the slip experimental material obtained from the aforementioned experiments is shown in Figure 6, and the material parameters were obtained as shown in Table 1.

**Table 1.** Material parameters obtained by slip experiment.

Material	Density (ton/mm^3^)	Yield stress (MPa)	Tensile strength (MPa)	Modulus (MPa)	Poisson ratio
Soluble material	$2.80\times 10^{-9}$	201	248	5615	0.25

### Establishment of the FEA model of the slip

#### FEA model

**Figure 7.**
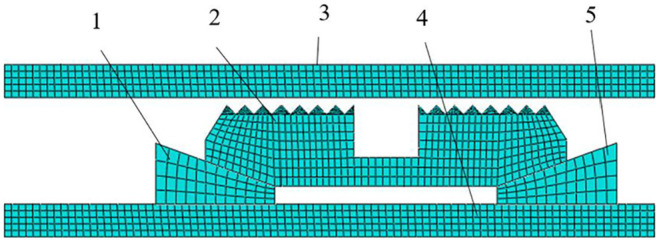
Grid model diagram of each component assembly. 1: lower cone; 2: slip; 3: casing; 4: central tube; 5: upper cone.

The components after simplification were meshed and assembled. All component mesh unit types were CPS4R, as shown in Figure 7.^21^ The analysis step was defined to show power analysis step.^22^ The normal contact surface of slip and sleeve was set to be “hard” contact, and the tangential contact between other components was a penalty contact.

#### Definition of material properties

Except for slip, the cross-section materials of other components could be used for reference to the existing engineering practice.^23^ The cone was 42CrMo, the casing was made of P110 steel, and the material parameters of slip were obtained from the slip body test. Mechanical properties are shown in Table 2.^24,25^

**Table 2.** Mechanical properties of each material.

Material	Density (ton/mm^3^)	Yield stress (MPa)	Tensile strength (MPa)	Modulus (MPa)	Poisson ratio
42CrMo	$7.85\times 10^{-9}$	950	1080	* $2.1\times 10^{5}$ *	* 0.28 *
P110	$7.85\times 10^{-9}$	760	893	* $2.0\times 10^{5}$ *	* 0.3 *

#### The settings of boundary conditions and load

**Figure 8.**
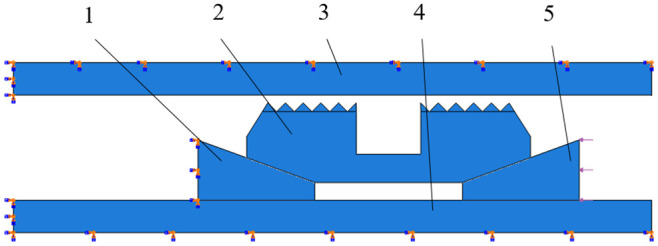
The diagram of part boundary conditions and load. 1: lower cone; 2: slip; 3: casing; 4: central tube; 5: upper cone.

In the actual working state, casing and central pipe after cementing must be completely constrained. The lower cone was threaded with the inner central tube and was also fully constrained. The upper cone acted as an active member in the anchoring mechanism, and the upper end portion was subjected to axial force transmitted by the rubber cylinder to generate a downward axial displacement. The slip was extended to the inner wall surface of the sleeve. Under the constraint of slip sleeve, the slip could only move along normal direction of the inner arc surface. The upper surface load of the upper cone was defined as 20 MPa. According to the aforementioned theory, boundary conditions and loads of each component were defined as shown in Figure 8.

#### Result of FEA calculation

**Figure 9.**
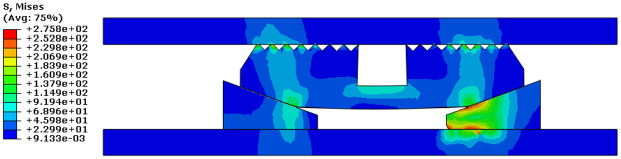
Von Mises stress cloud diagram after slip and casing occlusion.

**Figure 10.**
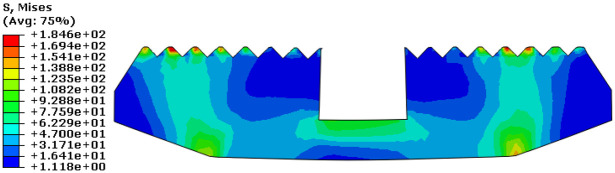
Von Mises stress cloud diagram of slip.

Taking a slip tooth number of 16, a tooth top angle of 90°, and a tooth spacing of 5 mm, we could observe Von Mises stress cloud diagram after slip and sleeve were engaged, as shown in Figures 9 and 10.

We obtained that the maximum stress appears on the surface of the cone in contact with slip and center tube, and the maximum stress value was 275.8 MPa. The maximum stress value of slip was 184.6 MPa, which appeared on the teeth of slip and sleeve. In the aforementioned figures, we found that the load distribution of the slip tooth surface was not uniform and there was stress concentration.

**Figure 11.**
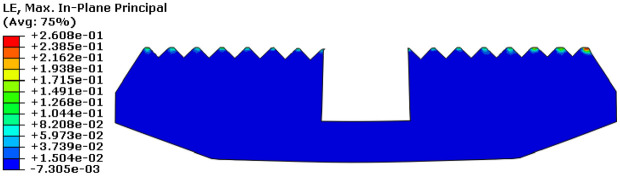
The true strain (LE) cloud diagram of slip.

**Figure 12.**
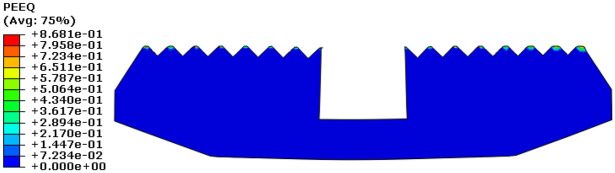
The equivalent plastic stain (PEEQ) cloud diagram of slip.

Figure 11 shows true strain (LE) cloud diagram. The maximum LE of slip was 0.2608, which appeared on the far right side of slip. Figure 12 shows equivalent plastic strain (PEEQ) cloud diagram. The maximum equivalent strain value of slip was 0.8681, which appeared on the slip tooth tip. The results indicated that the slip material has yielded.

**Figure 13.**
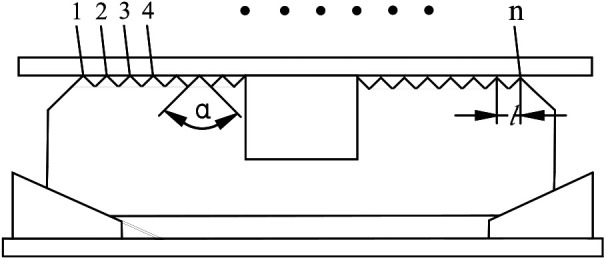
Physical meaning representation of various factors of slips.

This analysis was still considered to study pressure-bearing performance of slip under three influencing factors as shown in Figure 13. The factors were tooth number of slip (*n*), tooth top angle of slip (*α*), and tooth space of slip (*l*).

## Analysis and optimization of pressure-bearing performance of the slip

### Change rule of target parameters with the change in tooth number

The effects of Von Mises stress, LE, and PEEQ on the compressive properties of slips were analyzed when the tooth number was 12, 14, 16, 18, and 20, and the tooth top angle was fixed at 90°, and the tooth space was fixed at 5 mm. The 20 teeth of slip were numbered 1–20 from left to right, and other slips did not change the tooth number from middle to both sides.

**Figure 14.**
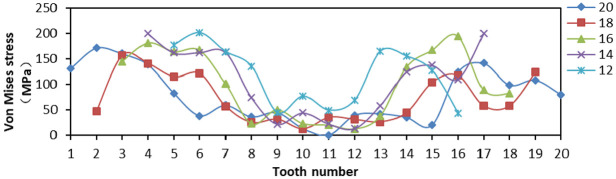
Relationship between Von Mises stress and tooth changes under different slips’ tooth number.

**Figure 15.**
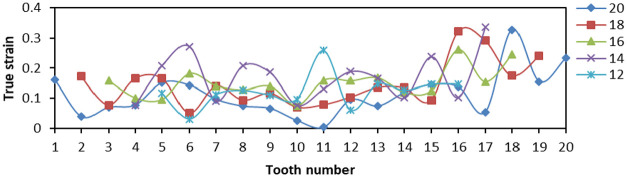
Relationship between true strain (LE) and tooth changes under different slips’ tooth number.

**Figure 16.**
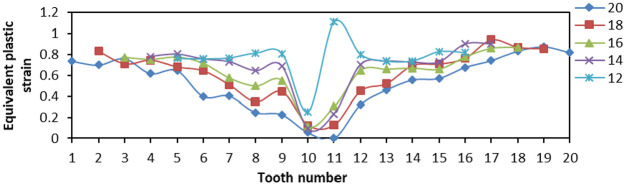
Relationship between equivalent plastic stain (PEEQ) and tooth changes under different slips’ tooth number.

From Figure 14, when tooth number was greater than 16, the distribution of Von Mises stress on slip tip was smaller, and the overall variation was smaller, then the force on slip teeth was more uniform. When the tooth number was less than 16, the Von Mises stress of outermost two teeth changes greatly, and the change amplitude was larger, which would increase the yield degree of single tooth. The LE of No. 16 tooth was smaller and lower than that of Nos 14, 18, and 20 teeth from Figure 15. From Figure 16, the PEEQ of No. 20 tooth was the smallest, and the strain of No. 12 tooth was the largest. We could obtain that the slip teeth had a larger deformation here. The more the number of slip teeth, the smaller the PEEQ.

From the stress and strain diagram above, the stress and strain values produced by teeth at both ends of slip were higher than those produced by teeth in the middle. When the tooth number was 14 and 16, the stress and strain values of each tooth were smaller, the amplitude was smaller, and the bending degree of slips was smaller.

### Change rule of target parameters with the change in tooth top angle

When tooth top angles were 70°, 80°, 90°, 100°, and 150°, respectively, the number of fixed teeth was 16 and the tooth spacing was 5 mm. The effects of Von Mises stress, LE, and PEEQ on the distribution of teeth were analyzed.

**Figure 17.**
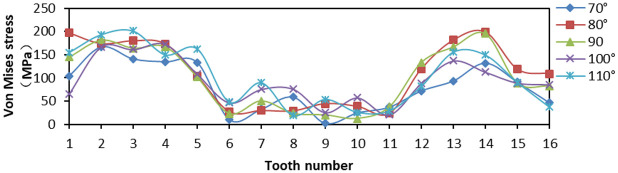
Relationship between Von Mises stress and tooth top angle changes under different slips.

**Figure 18.**
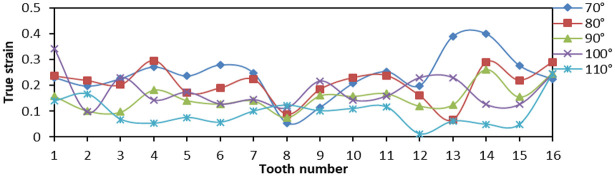
Relationship between true strain (LE) and tooth top angle changes under different slips.

**Figure 19.**
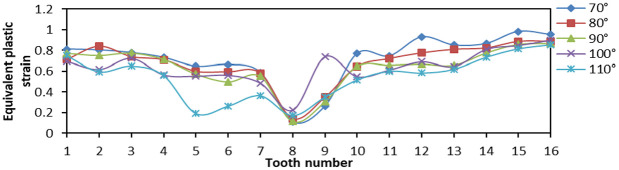
Relationship between equivalent plastic stain (PEEQ) and tooth top angle changes under different slips.

From Figure 17, when the tooth top angle was 80° and 90°, the Von Mises stress of tip of tooth distributes greatly at both ends of the slip, but the difference between two ends was small, and the overall amplitude was small. Figure 18 is a plot of the relationship between LE and tooth top angle changes under different slips, and the LE was the largest when tooth top angle was 70°. The LE was the minimum when tooth top angle was 110°, but its amplitude was larger. While the LE of 80° and 90° was lower and the amplitude was smaller, which was to reduce the local damage. From Figure 19, the PEEQ decreases step by step from 70° to 110°. When the tooth top angle was greater than 90°, the strain of each tooth was irregular and the yield point of strain decreases.

These figures illustrated that the smaller the tooth top angle of slip teeth, the higher the overall stress and strain, but the smaller the change in amplitude. Otherwise, the larger the tooth top angle, the smaller the overall strain and stress, but the great change in amplitude was not conducive to its compressive effect. According to the analysis and comparison, when the tooth top angle was 80° and 90°, the stress–strain value and variation amplitude of slip teeth were smaller as a whole, and the compressive effect was better.

### Change rule of target parameters with the change in tooth space

When tooth space was 4, 4.5, 5, 5.5, and 6, respectively, 16 teeth were fixed with 90° tooth top angle. The effects of contact stress and Von Mises stress distribution on the slip teeth were analyzed.

**Figure 20.**
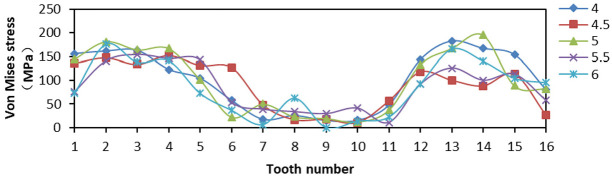
Relationship between Von Mises stress and tooth changes under different tooth space of slip.

**Figure 21.**
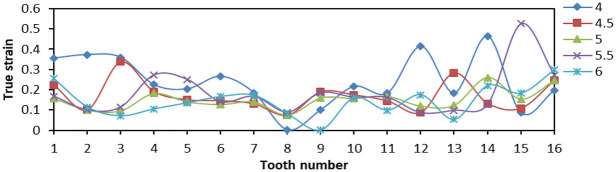
Relationship between true strain (LE) and tooth changes under different tooth space of slip.

**Figure 22.**
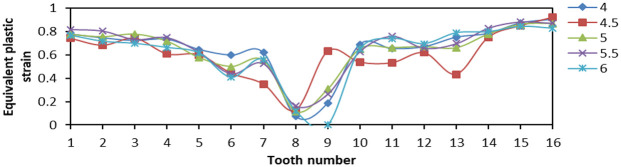
Relationship between equivalent plastic stain (PEEQ) and tooth changes under different tooth space of slip.

From Figure 20, the Von Mises stress values on both sides of tooth distribution were higher but the variation amplitude was smaller when the tooth space was 4 and 5 mm. When the tooth space was 4.5 and 5.5 mm, the difference in Von Mises stress value between two teeth was larger, and the overall amplitude was larger. When the tooth space was 6 mm, the stress distribution of two sides of tooth was not high, and the gap between two sides was small, the amplitude was small, and the overall stress situation was better. From Figure 21, when the space between teeth was 5.5 mm, the LE of No. 15 tooth appeared to be the maximum, that is, more than 0.5. When the tooth space was 6 mm, the LE was 0 at No. 9 tooth, although the LE distribution was small and uniform. Therefore, when the tooth space was 5 mm, the LE on the slip teeth was smaller and the amplitude was smaller, and the damage degree was lower. From Figure 22, the change in PEEQ of each slip was similar. When tooth space was 4.5 mm, the change in amplitude was large, which reduced the yield strength of individual teeth and was not conducive to compressive effect of slips.

From the aforementioned figures, we obtained that the smaller the tooth space, the greater the stress and strain. The larger the tooth space, the smaller the stress and strain. But the distribution amplitude was larger. Comparing the data changes, the stress and strain values of slip were smaller when the tooth space was 5 and 6 mm, and the amplitude was smaller, too. The result means that the slip has better bearing effect.

### Optimization of the tooth structure of the slip

Orthogonal design was used for overall design, comprehensive comparison, and statistical analysis. The selection is shown in Table 3.

**Table 3.** Structure parameter combination.

Combination	Number of teeth	Tooth top angle/°	Tooth space/mm
1	14	80	5
2	14	80	6
3	14	90	5
4	14	90	6
5	16	80	5
6	16	80	6
7	16	90	5
8	16	90	6

On the basis of distribution law of the aforementioned three influencing factors and the analysis results obtained from the aforementioned eight combinations of data, the variation amplitude of the distribution was similar to that of the previous one. Then, only the maximum Von Mises stress, the maximum LE, and the maximum PEEQ of each combination need to be compared.

**Figure 23.**
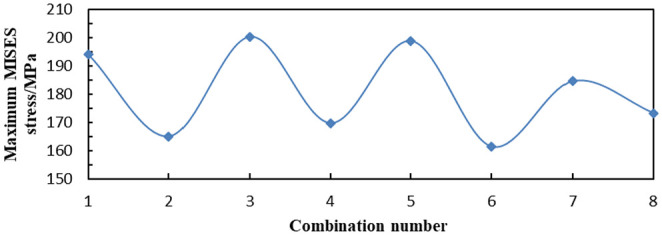
Maximum Von Mises stress of slip.

**Figure 24.**
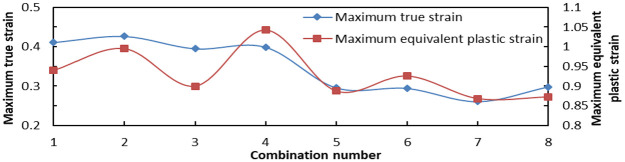
Maximum true strain (LE) and maximum equivalent plastic strain (PEEQ) of slip.

As shown in Figure 23, the maximum Von Mises stress of combinations 2 and 6 was the smallest, followed by 4, 7, and 8. In Figure 24, the maximum LE of No. 7 tooth was the smallest, followed by combinations 5, 6, and 8. Combinations 7 and 8 had the largest PEEQ, followed by 3, 5, and 6.

The comprehensive analysis showed that the maximum strain and the maximum Von Mises stress of the eight combinations (n=16,α=90°,l=6mm) were smaller, and the combination was the best combination of the pressure-bearing performance.

## Conclusion

The FEA method was used to analyze and optimize the pressure-bearing performance of compression packer. Finally, the following conclusions were drawn.

1. The distribution of stress and strain on the teeth of the slip was not uniform. The upper cone acted on the lower cone to make the slips slightly bend to interior, resulting in smaller stress and strain in the middle part of slips. The stress and strain on both sides showed obvious concentration. If the slip height was adjusted properly or the wedge angle of slips could be changed, the bending moment could be reduced.
2. The distribution of teeth in the slip had great influence on pressure-bearing performance of slip. The number of teeth was expressed in *n*. The tooth top angle was represented by α , and the tooth space was expressed in *l*. In the range of meeting 12≤n≤20,70°≤α≤110°,4mm≤l≤6mm , when *n* and *l* were bigger and α was smaller, the stress concentration became more obvious. When *n* and *l* were smaller and α was bigger, the stress and strain would increase. As a result, the number of teeth, the tooth top angle, and the tooth space were too large or too small, which was not conducive to its bearing effect.
3. Reasonable optimization of slip structure parameters could effectively improve the working life of slip. When the tooth space was *l* = 6 mm, the tooth top angle was *α* = 90, and the number of teeth was *n* = 16, load distribution of the slip was relatively uniform, the stress concentration was the smallest, and the self-damage was obviously reduced. This structure was a relatively optimal slip structure, which could provide reference for selecting the corresponding slip parameters in engineering practice.

Through analysis, the changing law of pressure-bearing capacity of the packer slips with structural parameters of the teeth was obtained. The best result of tooth space, tooth top angle, and tooth number was obtained. It was very important to improve the working performance and prolong the service life of the slip under the condition of reducing casing damage. It provided a theoretical basis for the study of casing and slips during the packer sealing process.

## Supplemental Material

SCI-19-0108_Editable_Graph – Supplemental material for Analysis of pressure-bearing performance and optimization of structural parameters of the slip in a compression packer Click here for additional data file. Supplemental material, SCI-19-0108_Editable_Graph for Analysis of pressure-bearing performance and optimization of structural parameters of the slip in a compression packer by Yang Tang, Peng Sun, Guorong Wang and Wang Li in Science Progress
